# Supplementary material for: What’s governance got to do with it? Examining the relationship between governance and deforestation in the Brazilian Amazon
Source: PLoS One. 2022 Jun 23;17(6):e0269729. doi: 10.1371/journal.pone.0269729 (PMC9223320; doi:10.1371/journal.pone.0269729)
Supplement: S1 Appendix — (DOCX) [file pone.0269729.s001.docx]

**S1 Appendix. Glossary.**

Master plan: Under article 182, it is mandatory for municipalities in Brazil with over 20,000 inhabitants to develop a plan which is a basic tool on development and urban sprawl policy. The documentation helps implement new principles of social function of property and establish a prevalence of diffuse rights over the individual property rights [1].

Agricultural sector: The number of employees in the livestock, forestry, fishing, and aquaculture sectors (Table 6449 - CEMPRE/IBGE).

Non-agricultural sector: The number of employees in manufacturing, construction, trade and repair of motor vehicles and motorcycles, accommodation and food, financial, insurance, and real estate sectors (Table 6449 - CEMPRE/IBGE).

Enterprise: A formally established industry with industrial activity as the main source of revenue that is registered in the National Registry of Legal Entities (CNPJ) of the Department of Federal Revenue. Enterprises with multiple economic activities or addresses are also counted, such as physical area (usually a continuous one) in which one or more economic activities are developed corresponding to a business address or a CNPJ code. (Annual survey of Industry- Enterprise - IBGE, available at: <https://www.ibge.gov.br/en/statistics/economic/industry-and-construction/16906-pia-enterprise-pia1.html?=&t=o-que-e>).

Zoning Law (*Lei sobre Zoneamento ou Uso e Ocupação do Solo*): Law specifying the use and occupation of urban land according to zones or delimited areas (Basic Municipal Information Survey - IBGE, available at: <https://www.ibge.gov.br/en/statistics/social/health/19143-survey-of-basic-municipal-information-editions.html?=&t=o-que-e>).

Division of Land Law (*Lei de Parcelamento do Solo*): Law that establishes the norms, standards, and criteria for new subdivision, dismemberments, and unification of urban land (Basic Municipal Information Survey - IBGE, available at: <https://www.ibge.gov.br/en/statistics/social/health/19143-survey-of-basic-municipal-information-editions.html?=&t=o-que-e>).

Urban Improvement Contribution Law (*Lei sobre Contribuição de Melhoria*): Law defining the tax charged from taxpayers benefited by public interventions and investments that result in the valuation of private properties. The law focuses on the appreciation of property ownership resulting from the performance of public works (Basic Municipal Information Survey - IBGE, available at: <https://www.ibge.gov.br/en/statistics/social/health/19143-survey-of-basic-municipal-information-editions.html?=&t=o-que-e>).

Urban Neighborhood Impact Law (*Lei sobre Estudo de Impacto de Vizinhança*): Law requiring technical studies prior to approval by the municipal authority of projects and activities. The law potentially affects the quality of life of the population living in the area and its surroundings (Basic Municipal Information Survey - IBGE, available at: <https://www.ibge.gov.br/en/statistics/social/health/19143-survey-of-basic-municipal-information-editions.html?=&t=o-que-e>).

Environmental Agency: Represents the executive branch of the Municipal Environmental System (*Sistema Municipal de Meio Ambiente*) and has the role of planning, coordinating, monitoring, and implementing the municipal environmental policy and agenda (Basic Municipal Information Survey - IBGE, available at: <https://www.ibge.gov.br/en/statistics/social/health/19143-survey-of-basic-municipal-information-editions.html?=&t=o-que-e>).

Environmental Fund: As part of the Municipal Environmental System, the Municipal Environmental Fund (*Fundo Municipal de Meio Ambiente*) may receive funds from the municipal budget or local environmental fees and taxes. The management board of the environmental fund comprises representatives of the local government and civil society that decide on the funding of environmental programs and projects (Basic Municipal Information Survey - IBGE, available at: <https://www.ibge.gov.br/en/statistics/social/health/19143-survey-of-basic-municipal-information-editions.html?=&t=o-que-e>).

Environmental Council: As part of the Municipal Environmental System, the environmental council (*Conselho Municipal de Meio Ambiente*) brings representatives of the public sector and civil society to deliberate and offer consulting for the municipal environmental agency regarding the design and implementation of local environmental policy (Basic Municipal Information Survey - IBGE, available at: <https://www.ibge.gov.br/en/statistics/social/health/19143-survey-of-basic-municipal-information-editions.html?=&t=o-que-e>).

Intermunicipal Consortium (*Consórcio Intermunicipal*): A formal agreement of cooperation between two or more municipalities in the same state to carry out activities of mutual interest that contribute towards the regional development (Basic Municipal Information Survey - IBGE, available at: <https://www.ibge.gov.br/en/statistics/social/health/19143-survey-of-basic-municipal-information-editions.html?=&t=o-que-e>).

Crop Density: Area of crop planted or intended for harvest in each year divided by the municipal area (square km) (Table 5457 - PPM/IBGE).

Cattle Density: Total count of livestock (bovines, equines, sheep, goats, chickens, pigs, fish, buffaloes, horses, quails) in each year divided by the municipal area (square km) (Table 3939 - PPM/IBGE).

**References**

1. da Silva JMP. Municipal and regional planning in Brazil: An overview of contemporary planning processes. 46th ISOCARP Congress. 2010.
