# Supplementary material for: What’s governance got to do with it? Examining the relationship between governance and deforestation in the Brazilian Amazon
Source: PLoS One. 2022 Jun 23;17(6):e0269729. doi: 10.1371/journal.pone.0269729 (PMC9223320; doi:10.1371/journal.pone.0269729)
Supplement: S1 Fig — (DOCX) [file pone.0269729.s002.docx]

**
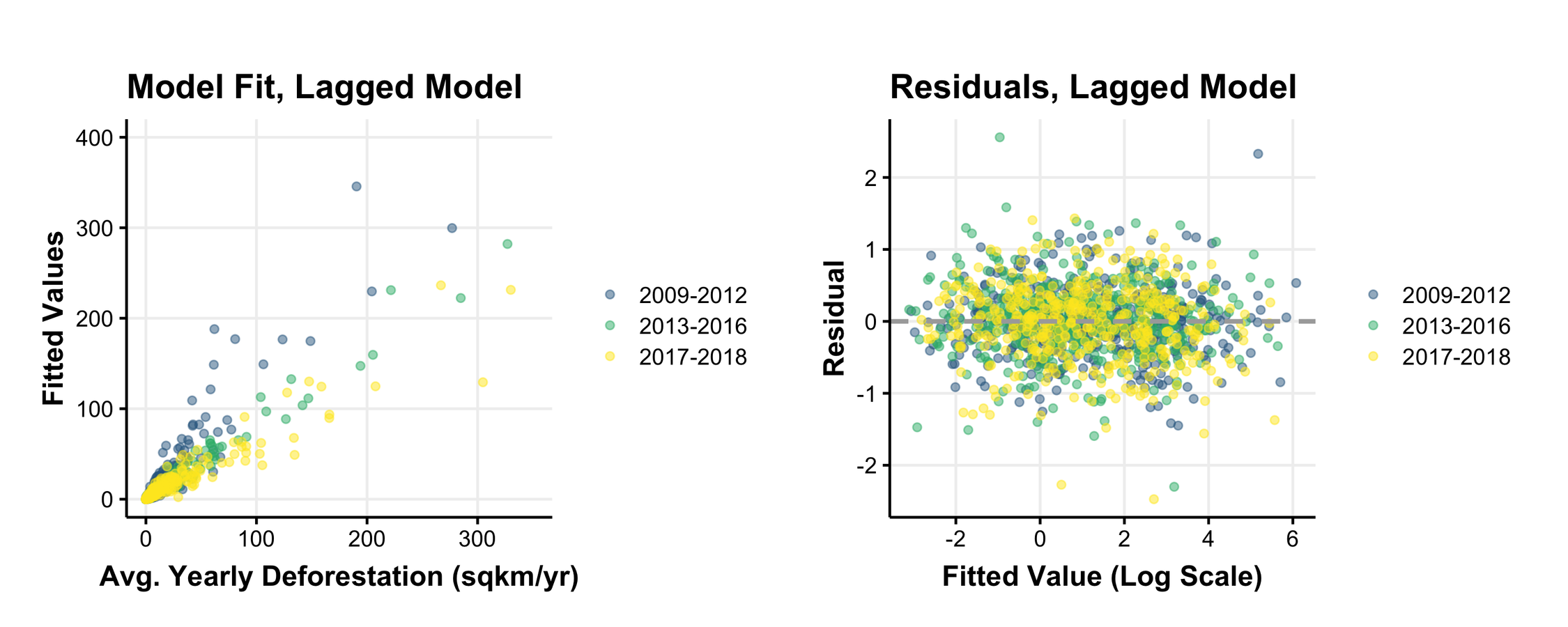
**

**S1 Fig. Model fit and residual plots for the lagged spatial panel regressions including all governance variables.**
