# Supplementary material for: What’s governance got to do with it? Examining the relationship between governance and deforestation in the Brazilian Amazon
Source: PLoS One. 2022 Jun 23;17(6):e0269729. doi: 10.1371/journal.pone.0269729 (PMC9223320; doi:10.1371/journal.pone.0269729)
Supplement: S2 Fig — Municipalities are colored based on the model residuals (difference between fitted and observed values) by time period. (DOCX) [file pone.0269729.s003.docx]

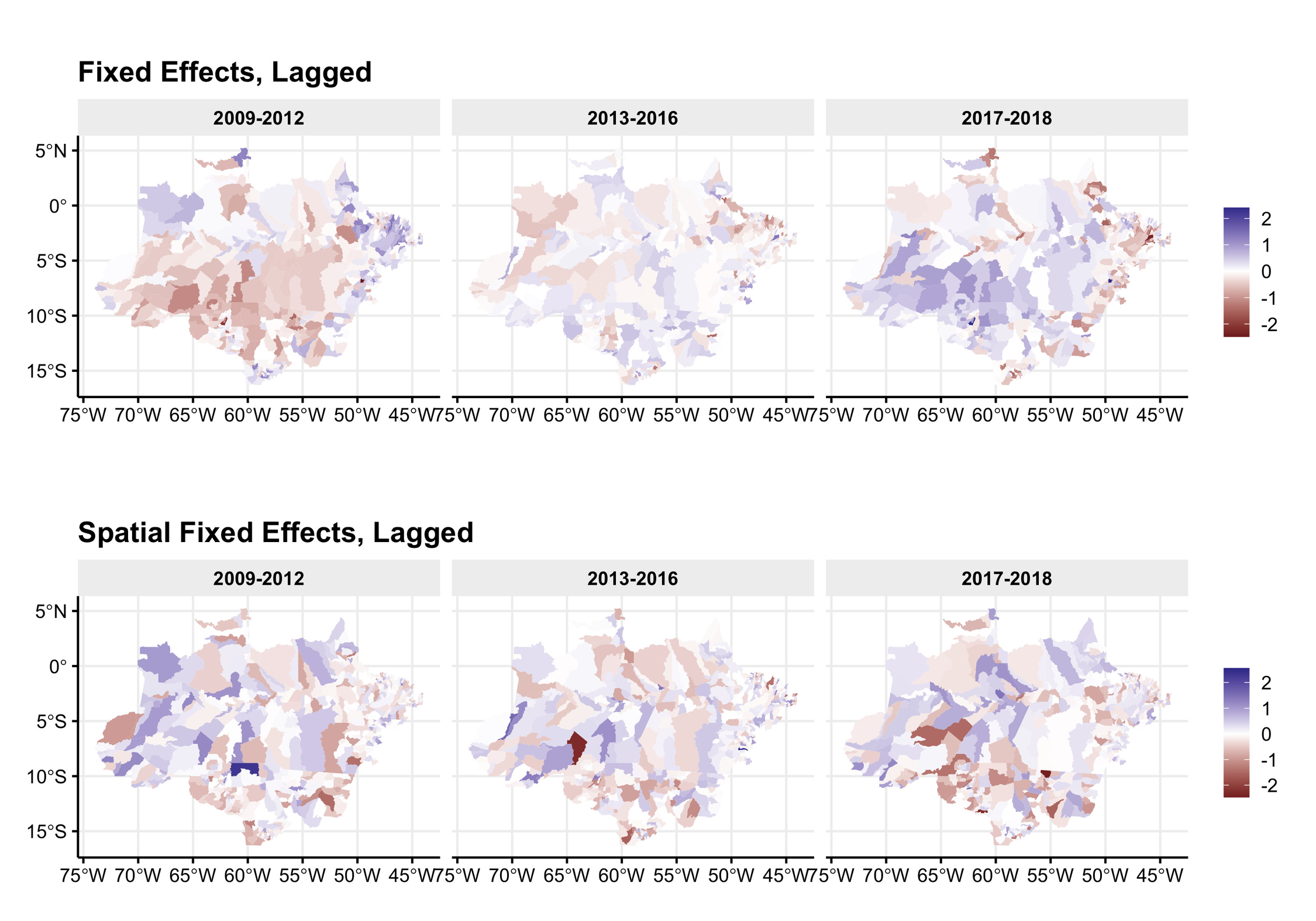


**S2 Fig. Maps of the model residuals for the non-spatial and spatial lagged panel regressions.** Municipalities are colored based on the model residuals (difference between fitted and observed values) by time period.
