# Supplementary material for: What’s governance got to do with it? Examining the relationship between governance and deforestation in the Brazilian Amazon
Source: PLoS One. 2022 Jun 23;17(6):e0269729. doi: 10.1371/journal.pone.0269729 (PMC9223320; doi:10.1371/journal.pone.0269729)
Supplement: S1 Text — (DOCX) [file pone.0269729.s004.docx]

## S1 Text. Spatial autocorrelation test.

To determine whether a spatial model specification was necessary, we computed the Moran’s I statistic on the residuals for both lagged and unlagged specifications (Fig S2), finding significant autocorrelation in all time periods 0.37, 0.24, 0.29 in the lagged model). Including a spatial error term reduced the residual autocorrelation in both models across all time periods (0.04, 0.05, 0.06 in the lagged model).
