# Supplementary material for: What’s governance got to do with it? Examining the relationship between governance and deforestation in the Brazilian Amazon
Source: PLoS One. 2022 Jun 23;17(6):e0269729. doi: 10.1371/journal.pone.0269729 (PMC9223320; doi:10.1371/journal.pone.0269729)
Supplement: S2 Text — (DOCX) [file pone.0269729.s005.docx]

## S2 Text. Alternate model specifications.

We evaluated the robustness of our model coefficients by specifying a series of reduced and alternate models, including a controls only model (Table S3), a significant variables only model (Table S4), and a model with variables from environmental governance and regulatory quality indicators (Table S5). We also provided the coefficient estimates for an unlagged model (Table S6), although we did not prefer this specification due to expected issues with endogeneity. With the exception of the variable representing presence of an environmental agency (loses statistical significance, Pr(>|t|) 0.13 and 0.18), all significant variables had stable coefficients and removing the other governance variables did not have a major effect on the estimated values.
