# Supplementary material for: What’s governance got to do with it? Examining the relationship between governance and deforestation in the Brazilian Amazon
Source: PLoS One. 2022 Jun 23;17(6):e0269729. doi: 10.1371/journal.pone.0269729 (PMC9223320; doi:10.1371/journal.pone.0269729)
Supplement: S3 Text — (DOCX) [file pone.0269729.s006.docx]

## S3 Text. Model comparison.

To measure relative model quality, we additionally calculated the Akaike Information Criterion (AIC) for each lagged model specification. The model including all governance variables slightly reduced model performance compared to other specifications, likely due to the fact that AIC calculations penalize models with additional terms and increasing model complexity. Including only the statistically significant governance variables from the full model slightly improved model performance relative to the controls-only models. Given the stable coefficients across most variables and our focus on model explanation rather than prediction, we chose to report the full model as our preferred specification.
