# Supplementary material for: What’s governance got to do with it? Examining the relationship between governance and deforestation in the Brazilian Amazon
Source: PLoS One. 2022 Jun 23;17(6):e0269729. doi: 10.1371/journal.pone.0269729 (PMC9223320; doi:10.1371/journal.pone.0269729)
Supplement: S1 Table — Only some of the reviewed sources were used in the final dataset. (DOCX) [file pone.0269729.s007.docx]

**S1 Table. All data sources reviewed for the study.** Only some of the reviewed sources were used in the final dataset.

| **Institution** | **Website revised** |
| --- | --- |
| IBAMA: Brazilian Institute of Environment and Renewable Natural Resources | <https://dadosabertos.ibama.gov.br/>,<https://servicos.ibama.gov.br/> |
| INPE: Instituto Nacional de Pesquisas Espaciais | <http://www.dgi.inpe.br/CDSR/> |
| IBGE: Instituto Brasileiro de Geografia e Estatística | <https://www.ibge.gov.br/pt/inicio.html> https://seriesestatisticas.ibge.gov.br/ <https://sidra.ibge.gov.br/> https://ww2.ibge.gov.br/ https://metadados.ibge.gov.br/ |
| Journal of Public Administration and Governance | [https://portal.fgv.br](https://portal.fgv.br/en) |
| DADOS: Brazilian Open Data Portal | <https://dados.gov.br/> |
| ImazonGEO: Geoinformação sobre a Amazónia | <https://imazongeo.org.br/> |
| IPEA: The Institute for Applied Economic Research | <https://www.ipeadata.gov.br/> |
| Tesoura Nacional: Ministry of Finance | <http://www.tesouro.fazenda.gov.br/> |
| Tesouro Transparente: Transparent Treasury Portal | <https://www.tesourotransparente.gov.br/> |
| CGU: Comptroller General of the Union | <https://www.portaldatransparencia.gov.br/> |
| Fala.BR: Plataforma Integrada de Ouvidoria e Acesso à Informação | <https://falabr.cgu.gov.br/> |
| AtlasBrasil: Atlas do Desenvolvimento Humano no Brasil | <http://www.atlasbrasil.org.br/> |
| ISA: Instituto SocioAmbiental | <https://www.socioambiental.org/pt-br> |
| Planet API | <https://www.planet.com/> |
| TSE: The Superior Electoral Court | <https://www.tse.jus.br/> |
| Terra-i org | <http://www.terra-i.org/> |
| GFW: Global Forest Watch | <https://www.globalforestwatch.org/map/> |
