# Supplementary material for: What’s governance got to do with it? Examining the relationship between governance and deforestation in the Brazilian Amazon
Source: PLoS One. 2022 Jun 23;17(6):e0269729. doi: 10.1371/journal.pone.0269729 (PMC9223320; doi:10.1371/journal.pone.0269729)
Supplement: S2 Table — (DOCX) [file pone.0269729.s008.docx]

**S2 Table. Model parameters for the all governance variables model with a lagged model specification.**

| **Variable** | **Estimate** | **Std. Error** | **t-value** | **Pr(>\|t\|)** |
| --- | --- | --- | --- | --- |
| Lagged deforestation | -0.18 | 0.03 | -6.34 | 0.00^***^ |
| Crop density | 0.01 | 0.01 | 1.73 | 0.08^*^ |
| Cattle density | -0.01 | 0.00 | -1.74 | 0.08^*^ |
| Population density | 0.00 | 0.00 | -0.92 | 0.36 |
| GDP | 0.00 | 0.00 | -0.14 | 0.89 |
| EG environmental council | 0.01 | 0.04 | 0.16 | 0.87 |
| EG environmental agency | 0.10 | 0.06 | 1.69 | 0.09 |
| EG environmental fund | -0.07 | 0.04 | -2.10 | 0.04^**^ |
| EG environmental employees | 0.01 | 0.01 | 0.82 | 0.41 |
| GE employees | 0.02 | 0.03 | 0.53 | 0.60 |
| GE intermunicipal consortiums | 0.03 | 0.04 | 0.69 | 0.49 |
| GE masterplan | 0.00 | 0.06 | -0.02 | 0.99 |
| ROL division of land | 0.04 | 0.04 | 1.07 | 0.29 |
| ROL urban improvement | 0.01 | 0.04 | 0.14 | 0.89 |
| ROL urban neighborhood | -0.03 | 0.04 | -0.79 | 0.43 |
| ROL zoning | -0.03 | 0.03 | -0.93 | 0.35 |
| RQ ag. companies | -0.05 | 0.04 | -1.23 | 0.22 |
| RQ non-ag. companies | -0.02 | 0.07 | -0.27 | 0.78 |
| RQ ag. employees | 0.06 | 0.03 | 2.17 | 0.03^**^ |
| RQ non-ag. employees | -0.08 | 0.04 | -1.90 | 0.06^*^ |
| RQ enterprise incentives | 0.00 | 0.03 | 0.04 | 0.97 |
| RQ enterprise restrictions | 0.04 | 0.04 | 0.99 | 0.32 |
| VA number of candidates | -0.02 | 0.02 | -1.30 | 0.19 |
| VA communication companies | -0.09 | 0.20 | -0.47 | 0.64 |
| VA proportion of votes | -0.01 | 0.02 | -0.75 | 0.45 |
| VA webpage | -0.03 | 0.03 | -0.76 | 0.45 |
| VA female mayor | -0.12 | 0.06 | -2.02 | 0.04^**^ |
| period 2009-2012 | -0.41 | 0.08 | -5.33 | 0.00^***^ |
| period 2013-2016 | -0.33 | 0.08 | -3.89 | 0.00^***^ |
| rho | 0.57 | 0.03 | 21.1 | 0.00^***^ |
| N | 1371 |  |  |  |
| ^***^p < 0.01, ^**^p < 0.05, ^*^p < 0.1 |  |  |  |  |
