# Supplementary material for: What’s governance got to do with it? Examining the relationship between governance and deforestation in the Brazilian Amazon
Source: PLoS One. 2022 Jun 23;17(6):e0269729. doi: 10.1371/journal.pone.0269729 (PMC9223320; doi:10.1371/journal.pone.0269729)
Supplement: S3 Table — (DOCX) [file pone.0269729.s009.docx]

**S3 Table. Model parameters for the controls only model with a lagged model specification.**

| **Variable** | **Estimate** | **Std. Error** | **t-value** | **Pr(>\|t\|)** |
| --- | --- | --- | --- | --- |
| Lagged deforestation | -0.18 | 0.03 | -6.41 | 0.00^***^ |
| Crop density | 0.02 | 0.01 | 1.96 | 0.05^*^ |
| Cattle density | -0.01 | 0.00 | -2.07 | 0.04^**^ |
| Population density | 0.00 | 0.00 | -0.51 | 0.61 |
| GDP | 0.00 | 0.00 | -0.45 | 0.65 |
| period 2009-2012 | -0.39 | 0.07 | -5.23 | 0.00^***^ |
| period 2013-2016 | -0.34 | 0.08 | -4.16 | 0.00^***^ |
| rho | 0.58 | 0.03 | 21.48 | 0.00^***^ |
| N | 1371 |  |  |  |
| ^***^p < 0.01, ^**^p < 0.05, ^*^p < 0.1 |  |  |  |  |
