# Supplementary material for: What’s governance got to do with it? Examining the relationship between governance and deforestation in the Brazilian Amazon
Source: PLoS One. 2022 Jun 23;17(6):e0269729. doi: 10.1371/journal.pone.0269729 (PMC9223320; doi:10.1371/journal.pone.0269729)
Supplement: S4 Table — (DOCX) [file pone.0269729.s010.docx]

**S4 Table.** **Model parameters for the significant variables only model with a lagged model specification.**

| **Variable** | **Estimate** | **Std. Error** | **t-value** | **Pr(>\|t\|)** |
| --- | --- | --- | --- | --- |
| Lagged deforestation | -0.18 | 0.03 | -6.39 | 0.00^***^ |
| Crop density | 0.01 | 0.01 | 1.74 | 0.08^*^ |
| Cattle density | -0.01 | 0.00 | -1.97 | 0.05^**^ |
| Population density | 0.00 | 0.00 | -0.81 | 0.42 |
| GDP | 0.00 | 0.00 | -0.27 | 0.79 |
| EG environmental agency | 0.09 | 0.06 | 1.52 | 0.13 |
| EG environmental fund | -0.07 | 0.03 | -2.20 | 0.03^**^ |
| RQ ag. employees | 0.05 | 0.03 | 1.97 | 0.05^**^ |
| RQ non-ag. employees | -0.08 | 0.04 | -1.99 | 0.05^**^ |
| VA female mayor | -0.11 | 0.06 | -1.93 | 0.05^*^ |
| period 2009-2012 | -0.41 | 0.07 | -5.47 | 0.00^***^ |
| period 2013-2016 | -0.33 | 0.08 | -4.01 | 0.00^***^ |
| rho | 0.58 | 0.03 | 21.28 | 0.00^***^ |
| N | 1371 |  |  |  |
| ^***^p < 0.01, ^**^p < 0.05, ^*^p < 0.1 |  |  |  |  |
