# Supplementary material for: What’s governance got to do with it? Examining the relationship between governance and deforestation in the Brazilian Amazon
Source: PLoS One. 2022 Jun 23;17(6):e0269729. doi: 10.1371/journal.pone.0269729 (PMC9223320; doi:10.1371/journal.pone.0269729)
Supplement: S5 Table — (DOCX) [file pone.0269729.s011.docx]

**S5 Table.** **Model parameters for the environmental governance and regulatory quality variables model with a lagged model specification.**

| **Variable** | **Estimate** | **Std. Error** | **t-value** | **Pr(>\|t\|)** |
| --- | --- | --- | --- | --- |
| Lagged deforestation | -0.18 | 0.03 | -6.36 | 0.00^***^ |
| Crop density | 0.01 | 0.01 | 1.78 | 0.08^*^ |
| Cattle density | -0.01 | 0.00 | -1.90 | 0.06^*^ |
| Population density | 0.00 | 0.00 | -0.74 | 0.46 |
| GDP | 0.00 | 0.00 | -0.10 | 0.92 |
| EG environmental council | 0.01 | 0.04 | 0.17 | 0.87 |
| EG environmental agency | 0.08 | 0.06 | 1.34 | 0.18 |
| EG environmental fund | -0.07 | 0.04 | -2.10 | 0.04^**^ |
| EG environmental employees | 0.01 | 0.01 | 0.85 | 0.39 |
| RQ ag. companies | -0.05 | 0.04 | -1.29 | 0.20 |
| RQ non-ag. companies | -0.01 | 0.06 | -0.12 | 0.90 |
| RQ ag. employees | 0.06 | 0.03 | 2.27 | 0.02^**^ |
| RQ non-ag. employees | -0.07 | 0.04 | -1.81 | 0.07^*^ |
| RQ enterprise incentives | 0.00 | 0.03 | 0.08 | 0.94 |
| RQ enterprise restrictions | 0.04 | 0.04 | 0.95 | 0.34 |
| period 2009-2012 | -0.43 | 0.08 | -5.68 | 0.00^***^ |
| period 2013-2016 | -0.36 | 0.08 | -4.34 | 0.00^***^ |
| rho | 0.58 | 0.03 | 21.46 | 0.00^***^ |
| N | 1371 |  |  |  |
| ^***^p < 0.01, ^**^p < 0.05, ^*^p < 0.1 |  |  |  |  |
