# Supplementary material for: What’s governance got to do with it? Examining the relationship between governance and deforestation in the Brazilian Amazon
Source: PLoS One. 2022 Jun 23;17(6):e0269729. doi: 10.1371/journal.pone.0269729 (PMC9223320; doi:10.1371/journal.pone.0269729)
Supplement: S6 Table — (DOCX) [file pone.0269729.s012.docx]

**S6 Table.** **Model parameters for the all governance variables model with an unlagged model specification.**

| **Variable** | **Estimate** | **Std. Error** | **t-value** | **Pr(>\|t\|)** |
| --- | --- | --- | --- | --- |
| Lagged deforestation | -0.09 | 0.02 | -3.88 | 0.00^***^ |
| Crop density | -0.01 | 0.01 | -0.81 | 0.42 |
| Cattle density | 0.00 | 0.00 | 1.49 | 0.14 |
| Population density | 0.00 | 0.00 | -0.48 | 0.63 |
| GDP | 0.00 | 0.00 | -0.09 | 0.93 |
| EG environmental council | -0.04 | 0.03 | -1.16 | 0.25 |
| EG environmental agency | 0.07 | 0.05 | 1.35 | 0.18 |
| EG environmental fund | 0.05 | 0.03 | 1.49 | 0.14 |
| EG environmental employees | 0.02 | 0.01 | 1.80 | 0.07^*^ |
| GE employees | -0.04 | 0.03 | -1.46 | 0.14 |
| GE intermunicipal consortiums | -0.02 | 0.04 | -0.67 | 0.50 |
| GE masterplan | 0.02 | 0.05 | 0.37 | 0.71 |
| ROL division of land | 0.08 | 0.03 | 2.24 | 0.03^**^ |
| ROL urban improvement | -0.04 | 0.03 | -1.20 | 0.23 |
| ROL urban neighborhood | -0.03 | 0.04 | -0.72 | 0.47 |
| ROL zoning | 0.01 | 0.03 | 0.20 | 0.84 |
| RQ ag. companies | 0.07 | 0.04 | 2.02 | 0.04^**^ |
| RQ non-ag. companies | 0.03 | 0.06 | 0.47 | 0.64 |
| RQ ag. employees | -0.03 | 0.02 | -1.34 | 0.18 |
| RQ non-ag. employees | -0.02 | 0.04 | -0.64 | 0.52 |
| RQ enterprise incentives | -0.01 | 0.03 | -0.29 | 0.77 |
| RQ enterprise restrictions | -0.01 | 0.03 | -0.17 | 0.86 |
| VA number of candidates | 0.00 | 0.02 | -0.09 | 0.93 |
| VA communication companies | -0.06 | 0.18 | -0.34 | 0.73 |
| VA proportion of votes | -0.01 | 0.02 | -0.62 | 0.54 |
| VA webpage | -0.04 | 0.03 | -1.39 | 0.16 |
| VA female mayor | 0.07 | 0.05 | 1.41 | 0.16 |
| period 2009-2012 | -1.07 | 0.10 | -11.23 | 0.00^***^ |
| period 2013-2016 | -1.42 | 0.11 | -13.24 | 0.00^***^ |
| rho | 0.72 | 0.02 | 33.80 | 0.00^***^ |
| N | 1371 |  |  |  |
| ^***^p < 0.01, ^**^p < 0.05, ^*^p < 0.1 |  |  |  |  |
