# Supplementary material for: What’s governance got to do with it? Examining the relationship between governance and deforestation in the Brazilian Amazon
Source: PLoS One. 2022 Jun 23;17(6):e0269729. doi: 10.1371/journal.pone.0269729 (PMC9223320; doi:10.1371/journal.pone.0269729)
Supplement: S7 Table — (DOCX) [file pone.0269729.s013.docx]

## S7 Table. Akaike Information Criterion across model specifications and predictor subsets.

| **Model Specification** | **AIC** |
| --- | --- |
| All Governance Variables, Lagged | 8632.102 |
| Controls Only, Lagged | 8616.36 |
| Significant Governance Variables, Lagged | 8607.231 |
| EG and RQ Variables, Lagged | 8617.15 |
